# Impact of vitamin D and vitamin D receptor on the trophoblast survival capacity in preeclampsia

**DOI:** 10.1371/journal.pone.0206725

**Published:** 2018-11-08

**Authors:** Martina Hutabarat, Noroyono Wibowo, Barbara Obermayer-Pietsch, Berthold Huppertz

**Affiliations:** 1 Postgraduate Department, Doctorate Program Faculty of Medicine, University of Indonesia, Jakarta, Indonesia; 2 Department of Obstetric and Gynecology, Division of Maternal Fetal Medicine, Faculty of Medicine, University of Indonesia, Jakarta, Indonesia; 3 Department of Internal Medicine, Division of Endocrinology and Diabetology, Medical University of Graz, Graz, Austria; 4 Department of Cell Biology, Histology and Embryology, Gottfried Schatz Research Center, Medical University of Graz, Graz, Austria; Xavier Bichat Medical School, INSERM-CNRS - Université Paris Diderot, FRANCE

## Abstract

**Background:**

Preeclampsia and intra-uterine growth restriction (IUGR) are major health problems during pregnancy affecting both mother and child. Defective placental development and failure of trophoblast differentiation during pregnancy are important aspects in the pathogenesis of both syndromes. Recent studies have shown that autophagy is involved in the trophoblast survival capacity. As vitamin D has a central role in many cellular processes, we studied the relation of vitamin D and autophagy in those processes of preeclampsia and IUGR.

**Methods:**

Serum and placental samples from four groups of cases; normal term, IUGR, early-onset and late-onset preeclampsia, were analyzed for 25(OH)D vitamin D, sFLT1, PGF, LGALS13 in serum and vitamin D receptor (VDR), MAP1LC3B and BECN1 in placental tissues.

**Results:**

There was a significant difference in the sFLT1/PGF ratio in preeclamptic cases compared to controls and IUGR. There was a significant difference between these groups in the MAP1LC3B/BECN1 ratio as marker of the trophoblast survival capacity with a significantly reduced ratio in villous trophoblast of early-onset preeclampsia. Maternal vitamin D deficiency was found in all pathological pregnancies combined with significantly reduced staining levels of placental VDR in IUGR. Finally, there was a strong and significant negative correlation between the survival capacity (MAP1LC3B/BECN1) and both maternal vitamin D and placental VDR in the preeclampsia groups.

**Conclusion:**

Vitamin D and intracellular VDR are strongly related to the trophoblast survival capacity in preeclampsia.

## Introduction

Preeclampsia is a severe pregnancy-associated syndrome, leading to morbidity and mortality of both mother and child worldwide.[1–4] The placenta as a specific organ of pregnancy has been considered to be essentially involved in the pathogenesis of preeclampsia.[5] Placentation defects in the very early stages of pregnancy are thought to be the main predisposing factors for preeclampsia, while the severity of the disease is determined by the maternal response to toxic factors of the placenta.[5,6]

Placentation defects in early pregnancy may result in inadequate trophoblast differentiation followed by subsequent impairment of villous maturation and villous trophoblast turnover.[7] The dysregulation of villous trophoblast during preeclampsia can be followed using placental protein 13 (PP13, LGALS13) as a placenta-specific marker released into maternal blood. [8] This complex process and its cascade of events may induce endothelial dysfunction and systemic inflammation of the mother.[1] Endothelial dysfunction during preeclampsia can be visualized using (anti-) angiogenic markers such as sFLT1 and PGF. [9]

On the cellular level, cells have developed the ability to maintain their integrity to environmental stress activating the process of autophagy.[10–12] Failure of finalizing the autophagy process can be identified by changes in the ratio of the autophagy markers, microtubule associated protein 1, light chain 3 beta (MAP1LC3B) and beclin-1 (BECN1), (MAP1LC3B/BECN1). If a cell fails to maintain its integrity, the cell tends to enter into the processes of cell death, ranging from apoptosis to necrosis. Hence, the ratio of MAP1LC3B/BECN1 does not only show the failure of autophagy but also gives an idea on the survival capacity of a cell.[13]

Nutrition is a main regulator of autophagy.[14,15] Vitamin D and the associated cellular signaling components, such as the vitamin D receptor (VDR), are molecules that are highly related to the autophagy process. Vitamin D plays a central role in many cellular processes such as cellular proliferation and differentiation, and immunometabolism either via genomic or nongenomic actions.[16] The involvement of vitamin D in basic biological processes such as autophagy has led to a variety of research questions.[17–19] At the same time, recent data provide evidence that in South East Asian countries including Indonesia vitamin D insufficiency is a major issue. One of the reasons discussed was that only about half of the children in Indonesia consumed dairy products on a daily basis. [20] Therefore, the aim of our study was to define the role of vitamin D in processes of cellular survival in the villous trophoblast of the human placenta in pregnancy pathologies such as preeclampsia and fetal growth restriction.

## Material and methods

This study included pregnant women attending the Cipto Mangunkusumo Hospital and Budi Kemuliaan Hospital in Jakarta, Indonesia from August to October 2015. We assigned the women and their respective samples to four groups: normal gestation with term delivery, late-onset preeclampsia (LoPE), early-onset preeclampsia (EoPE), and intrauterine growth restriction (IUGR). We defined the inclusion criteria to include all pregnant women whose pregnancy was terminated by cesarean section due to early and late-onset preeclampsia, IUGR and normal term pregnancy without complications in the labor process. We excluded all pregnant women who suffered from other diseases, such as heart disease in pregnancy, diabetes mellitus, auto-immune diseases during pregnancy such as lupus erythematosus, pregnant women with congenital abnormalities of the baby, preterm labor, severe infection during pregnancy or women not willing to take part in this study. We applied the mean difference formula in each group to determine the sample size, which was 10 samples for each group. Hence, the total number of subjects in this study was 40 with 10 subjects in the normal gestation group, 11 subjects with late-onset preeclampsia, 9 subjects with early-onset preeclampsia, and 10 subjects in the IUGR group.

The subjects were pregnant women whose delivery dates were based on obstetric medical indications. We defined normal gestation as pregnancy until term without any complications and based the definition of preeclampsia on the 2013 ACOG criteria [21], including blood pressure of ≥140/90 mmHg with proteinuria after 20 weeks of pregnancy in previously normotensive women. Early-onset preeclampsia (EoPE) was defined as preeclampsia diagnosed before 34 weeks, while late-onset preeclampsia (LoPE) was defined as preeclampsia diagnosed at or after 34 weeks of pregnancy.[22,23] The major difference between early and late-onset preeclampsia is that early-onset preeclampsia is usually complicated by intrauterine growth restriction and/or other organ involvement with severe clinical features.[21,23] Intrauterine growth restriction (IUGR) was defined as fetal weight lower than the 10^th^ percentile on the growth chart. [24,25]

In this study we collected samples from maternal serum, cord venous serum and placental tissues. The amount of 10 ml maternal serum was collected at the time of patient admission in the delivery or emergency room. In the labor process after the baby was delivered, 10 ml cord venous serum was collected and followed by collection of placental tissues. We processed blood serum samples immediately by standardized preanalytical procedures and stored them at -80°C, while we processed paraffin blocks of the placental tissues according to a standardized pathology protocol. We examined the factors sFLT1, PGF and placental protein 13 (LGALS13) using maternal serum, while we evaluated 25(OH)vitamin D levels in maternal serum and cord venous serum. For the measurements of the autophagy proteins MAP1LC3B and BECN1 and the vitamin D receptor we used placental tissues.

At the Endocrinology Lab Platform at the Medical University of Graz, Austria, we performed the laboratory measurements of angiogenic factors (sFLT1 and PGF), LGALS13 and 25(OH)D levels. We assessed placental tissues at the Department of Cell Biology, Histology and Embryology at the Medical University of Graz, Austria.

We measured the angiogenic factors sFLT1 and PGF using the Elecsys equipment (Roche Diagnostics, GmbH, Vienna Austria). Sample volume was 20 μl for sFLT1 and 50 μl for PGF, range 10–85,000 ng/ml for sFLT1 and 3–10,000 mg/ml for PGF. The sensitivity was <15 pg/ml for sFLT1 and 10 pg/ml for PGF. The intra-assay variation coefficient was <2% for both sFLT1 and PGF, the inter-assay variation coefficient was 2.6–30% for sFLT1 and 2.0–2.4% for PGF.

We measured placental protein 13 (LGALS13) concentrations utilizing a solid-phase sandwich ELISA with a pair of LGALS13 specific monoclonal antibodies (Hylabs, Israel), marked with amplified biotin-extravidin-horseradish-peroxidase complex and developed with tetramethylbenzidene substrate. Using the optical density at 450 nm against a 650-nm background we translated the optical measurements into a quantitative amount using a calibration curve made of recombinant LGALS13 standards (0–500 pg/ml).

We assessed serum vitamin D (25(OH)D) levels of maternal and cord blood by automated chemiluminescence immunoassays on the iSYS automated system (Immunodiagnostic Systems, Boldon, UK, with an intraassay and interassay coefficient of variation of 6.2% and 11.6%, respectively).

In this study, we detected the autophagy markers MAP1LC3B and BECN1 and the vitamin D receptor (VDR) using immunohistochemistry. We mounted five (5) μm thick formalin-fixed paraffin-embedded tissue sections on Superfrost Plus slides (Menzel/Thermo Fisher Scientific). According to the standard procedures, we deparaffinized sections in Histolab Clear (Sanova) and rehydrated them through a mixture of 100% ethanol/Histolab Clear 1:1, followed by a series of graded alcohol and finally aqua dest.

We performed antigen retrieval for 7 min at 120°C in a decloaking chamber (Biocare Medical) in 10 mM sodium citrate buffer pH 6.0 for the anti-VDR antibody (abcam) and the anti-BECN1 antibody (abcam) or Epitope Retrieval Solution pH 8.0 (Novocostra, Leica) for the MAP1LC3B antibody (Novus biologicals). After 20 min cooling at room temperature, we transferred slides in TBS including 0.05% Tween 20 (TBS/T, Merck). We performed staining of sections either manually or using a staining robot (Autostainer 360, Thermo Fisher Scientific). To quench endogenous peroxidase, we incubated slides with hydrogen peroxide block (Lab Vision/Thermo scientific) for 10 min and after washing with TBS/T, we blocked nonspecific background by incubation with Protein Block (Lab Vision/Thermo scientific) for 7 min. We diluted primary antibodies in Antibody Diluent (Dako) as follows; VDR 1:700, BECN1 1:280, MAP1LC3B 1:3000, and incubated sections for 45 min at room temperature. After washing with TBS/T, we incubated slides for 20 min with the UltraVision HRP-labelled polymer and following another washing step, we visualized the polymer complex by incubating the slides with AEC Chromogen Single Solution (Lab Vision/Thermo scientific) for 10 min. Following washing steps with aqua dest, we counterstained sections with hemalaun and mounted them with Kaiser’s glycerin gelatine (Merck) or Aquatex (Merck).

We assessed placental tissues on 3 blocks per placenta, taken from central, intermediate and peripheral positions of a placenta. We quantified staining of images with a Leica DM 6000B microscope with an Olympus DP 72 camera and VIS visiopharm software (Visiopharm, Denmark), based on systematic uniform random sampling. For each slide, the software chose 10 pictures randomly and systematically and placed a point grid with 16 times 12 points on each picture (newCAST software, Visiopharm). Each point was assigned to a specific category. The categories for MAP1LC3B and BECN1 were: intervillous space, villous stroma, villous trophoblast and fibrin. Due to the location of VDR within nuclei, the categories for the vitamin D receptor were: intervillous space, villous stroma, trophoblast nuclei and fibrin. Within the categories ‘villous trophoblast’ and ‘trophoblast nuclei’ staining intensity was ranked 3 for the strongest staining, 2 for moderate staining, and 1 for the weakest staining or 0 if the dot was placed on trophoblast without staining.

We calculated the ratio between strong/moderate and weak/no staining (also called positivity) by counting the dots with ranks 2 and 3 divided by the sum of the dots with ranks 0 and 1 within villous trophoblast/trophoblast nuclei and called this ratio ‘positivity’. We quantified total staining in the trophoblast as follows (called ‘stained trophoblast’): dots in villous trophoblast/trophoblast nuclei were ranked 0 to 3 and for each protein and image, and the ranking was added and divided by the total number of dots in villous trophoblast/trophoblast nuclei. Finally, we calculated the ratio of MAP1LC3B to BECN1 using the two above approaches to calculate staining intensity parameters. For each parameter, we divided the two values of MAP1LC3B and BECN1 resulting in the ratio of MAP1LC3B/BECN1. This ratio represents one indicator of autophagy limitations in a cell, as it shows the cellular survival mechanism as one of the aspects of autophagy.

We tabulated, edited and coded all data based on the purpose of the study and statistically analyzed the data using R Statistics Software version 3.4.0 for Windows operating system. We used the univariate analysis for descriptive characteristics, and assessed differences between autophagy markers and nutritional status between each group by bivariate One-way ANOVA or Kruskal-Wallis test. Correlations between variables were calculated by Spearman’s correlation tests.

Before the start of the study, signed informed consent was given to the research subject and her family and ethical clearance was given from The Health Research Ethic Commitee—Faculty of Medicine University of Indonesia and the Cipto Mangunkusumo Hospital (*Komite Etik Fakultas Kedokteran Universitas Indonesia* No:668/UN2.F1/ETIK/2015) as approval.

## Results

### Characteristics

Forty pregnant women participating in this study had a mean age of 30.4 ± 5.9 years (standard deviation (SD)). Twenty-three women (57.5%) were between 25–34 years, 12 women (30%) aged above 35 years, and 5 women (12.5%) aged under 24 years.

The median gestational age of the subjects was 38 weeks with the lowest gestational age of 25 and highest gestational age of 40 weeks. There was a statistically significant difference in gestational age of the mothers particularly in the early-onset preeclampsia group (p<0.001).

Anthropometric measurements showed a mean body mass index (BMI) before pregnancy was 23.4 kg/m^2^ ± 4.7 kg/m^2^. There were no significant differences in anthropometric variables between the four groups. The results are depicted in Table 1.[13]

**Table 1:** Subjects’ characteristics.

Characteristics	Group					
	Normal	Late-Onset PE	Early-Onset PE	IUGR	Total	p-value
	(n = 10)	(n = 11)	(n = 9)	(n = 10)	(n = 40)	
Age (years)	32.3 (5.5)	29.4 (6.6)	32.6 (5.3)	27.8 (5.4)	30.4 (5.9)	0.209^#^
**GA, range (weeks)**	**39 (38; 40)**	**38 (36; 40)**	**32 (25; 35)**	**37 (31; 38)**	**38 (25; 40)**	**<0.001**^$^
Parity (n)						
Primi	5	4	4	6	19	0.780*
Multi	5	7	5	4	21	
BMI before pregnancy (kg/m^2^)	24.3 (2.8)	23.7 (5.4)	24.9 (6.3)	20.7 (2.9)	23.4 (4.7)	0.205^#^
**BP, range (mmHg)**						
**Systolic**	**110 (100;140)**	**150 (140; 190)**	**150 (140; 240)**	**110 (100; 140)**	**140 (100; 240)**	**<0.001**^$^
**Diastolic**	**70 (60; 80)**	**100 (90; 140)**	**100 (90; 160)**	**70 (70; 90)**	**90 (60; 160)**	**<0.001**^$^
**Baby birth weight (g)**	**3,267.0 (354.5)**	**2,972.7 (586.7)**	**1,337.2 (640.6)**	**1,933.5 (437.2)**	**2,418.5 (920.3)**	**<0.001**^#^
Baby BW below 10^th^ percentile (n)	0	3	3	10	16	

Numeric data is shown as mean (standard deviation) or median (min; max); GA = Gestational Age; BW = body weight; BMI = Body Mass Index; BP = Blood Pressure; PE = preeclampsia; IUGR = Intrauterine Growth Restriction; SD = Standard Deviation;

^#^One-wayANOVA Test;

^$^Kruskal-Wallis Test.

This table has already been published in [13] but is shown again for a better appreciation of the study cohort.

### Defective placentation and survival capacity

There was a significant difference of the sFLT1/PGF ratio between normal and pathological pregnancy cases. In addition, a post hoc analysis showed significant differences of LGALS13 levels between the normal pregnancy and the IUGR group (p = 0.022) as shown in Table 2.

**Table 2:** Angiogenic factors and LGALS13 test results.

Variable	Group				p-value
	Normal	Late-Onset PE	Early-Onset PE	IUGR	
	(n = 10)	(n = 7)	(n = 8)	(n = 10)	
Ratio sFLT1/PGF	10 (3.9; 48.5)^a^^,^^b^	83.8 (11.9; 328.2)^a^	226.5 (55.8; 2427.0)^b^^,^^c^	31.2 (4.3; 183.0)^c^	**<0.001**^#^
LGALS13 (pg/ml)	512.4 (246.7; 4701.0)^d^	419.0 (1.0; 1090)	367.6 (120.0; 931.7)	253.8 (46.2; 611.7)^d^	**0.050**^#^

Numeric data is shown by mean (standard deviation) or median (min; max);

^#^One-way ANOVA test

^a^: post hoc analysis between normal and late PE (p = 0.022);

^b^: post hoc analysis between normal and early PE (p<0.001);

^c^: post hoc analysis between early PE and IUGR (p = 0.013);

^d^: post hoc analysis between normal and IUGR (p = 0.022)

For stained trophoblast, there were significant differences in autophagic survival markers (p = 0.024, one-way ANOVA test), the MAP1LC3B/BECN1 ratio, as assessed by immunohistochemistry of villous trophoblast in the four groups.[13] There were higher mean values of the MAP1LC3/BECN1 ratio in normal pregnancy (1.63±0.34) and late-onset preeclampsia (1.46±0.41) compared to the early-onset preeclampsia (1.13±0.20) and IUGR (1.39±0.34) groups.[13] The early-onset preeclampsia group had the lowest ratio among the groups and showed a significantly lower ratio than the control group (p = 0.008, post-hoc test). Similarly, in the parameter of positivity the normal pregnancy group showed much higher values compared to the other three groups, but the values did not reach significance (p = 0.169, Kruskal-Wallis test).[13]

Measurements of the cell autophagic survival markers MAP1LC3B/BECN1 were also performed in relation to gestational age. There was no significant difference of staining level related to gestational age; however, distribution for each subject in all pregnancy groups showed that subjects in the early-onset preeclampsia group and IUGR group had lower number of MAP1LC3B/BECN1 ratios compared to the other two groups. This study cannot tell whether this was due to early-onset preeclampsia/IUGR or gestational age below 35 weeks.[13]

### Vitamin D in pregnancy

The analysis of vitamin D status included 25(OH)vitamin D (25(OH)D) levels in maternal blood, umbilical cord blood, and vitamin D receptor (VDR) in placental tissues.

There was a significant difference in median 25(OH)D levels between groups. While 25(OH)D vitamin D levels in the normal pregnancy group were in the normal range, the early-onset preeclampsia group had 25(OH)D deficiency. Also the late-onset preeclampsia and IUGR groups showed 25(OH)D insufficient level based on reference values for normal and deficient vitamin D levels in maternal blood of >32 ng/ml and <20 ng/ml.[26,27] Post hoc analysis showed significant differences between normal pregnancy and all disease groups.

There was a significant difference of cord blood 25(OH)D level between groups, in which IUGR had the lowest level. Post hoc analysis showed a significant difference between normal pregnancy and the late onset preeclampsia group (p = 0.009) as well as the IUGR group (p = 0.004) as shown in Table 3.

**Table 3:** Vitamin D (25(OH)D) status in the four groups.

Variable	Group				p-value
	Normal	Late Onset PE	Early Onset PE	IUGR	
	(n = 10)	(n = 11)	(n = 9)	(n = 10)	
Maternal (ng/dL)	36.6 (23.8; 61.4)^a^^,^^b^^,^^c^	26.0 (11.3; 38.9)^a^	18.0 (10.3; 44.5)^b^	20.2 (13.5; 29.7)^c^	**0.007**^$^
Cord (ng/dL)	35.0 (19.1; 51.0)^d^^,^^e^	21.4 (7.0; 28.2)^d^	16.5 (11.3; 46.7)	15.4 (14.4; 26.3)^e^	**0.005**^$^

Data are shown as mean (standard deviation) or median (min;max);

^#^One-way ANOVA Test;

^$^Kruskal-Wallis Test

^a^: Post hoc test between normal pregnancy and late PE (p = 0.066)

^b^: Post hoc test between normal pregnancy and early PE (p = 0.022);

^c^: Post hoc test between normal pregnancy and IUGR (p = 0.004)

^d^: Post hoc test between normal pregnancy and late PE (p = 0.009);

^e^: Post hoc test between normal pregnancy and IUGR (p = 0.004)

### Placental vitamin D receptor (VDR)

**Fig 1:**
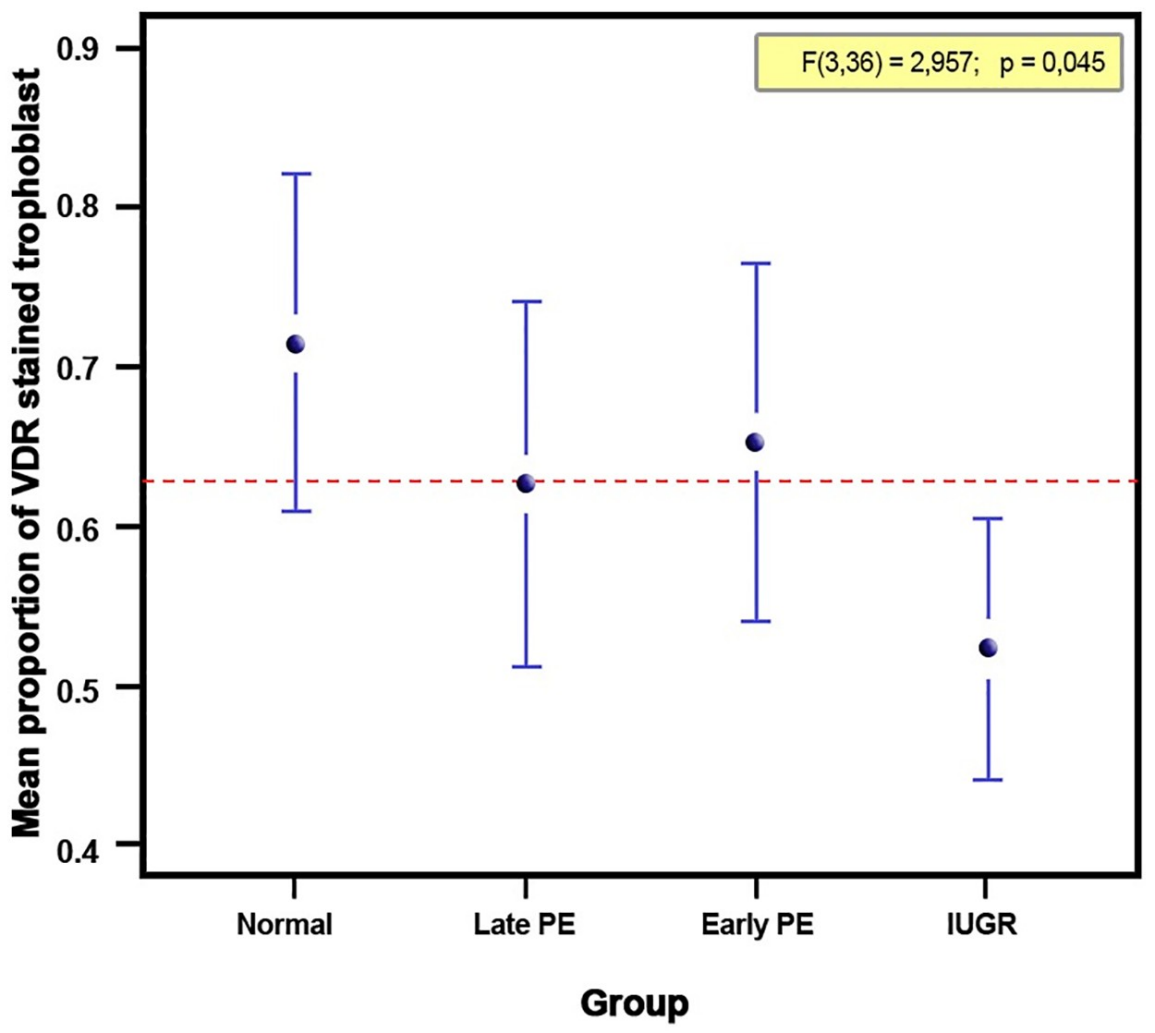
Placental VDR staining level in the four groups. There was a significant difference in the stained trophoblast proportion of VDR between groups (p = 0.045). Post hoc analysis identified that placental VDR of the normal pregnant group was significantly higher compared to the IUGR group (p = 0.029).

**Fig 2:**
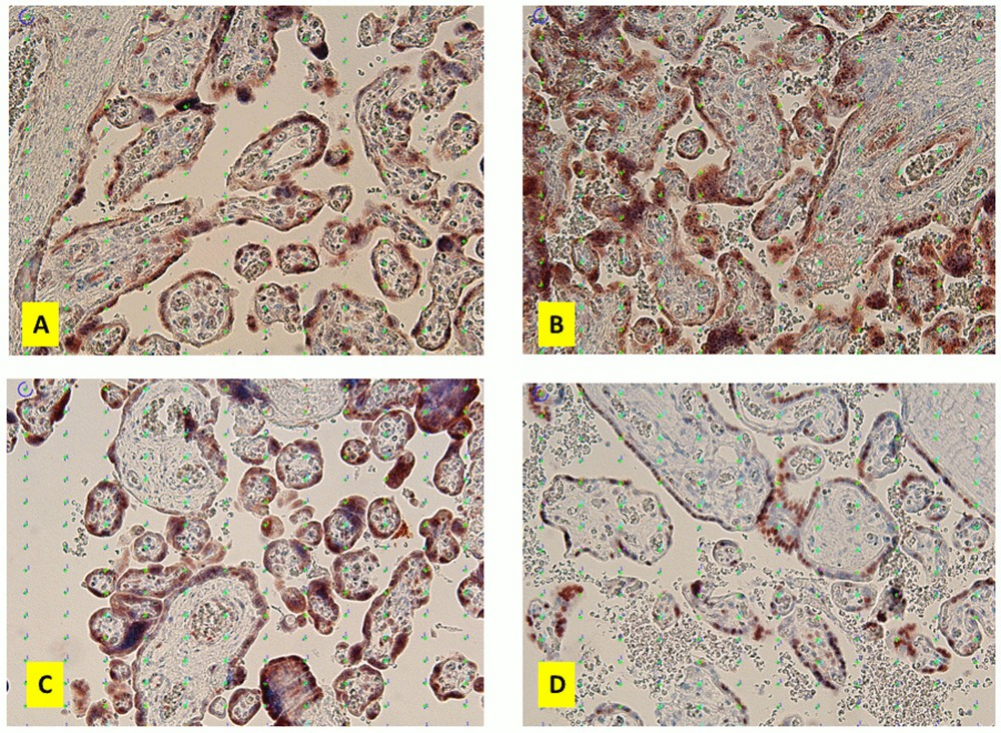
Images of VDR staining in the four groups. Placental immunohistochemical staining for VDR in normal term pregnancy (A), late-onset preeclampsia (B), early-onset preeclampsia (C) and IUGR (D). Quantification was performed based on systematic random sampling. The point grid with 16 times 12 points placed on each image was used for systematic random quantification of staining. There was stronger staining for VDR in normal term pregnancy compared to other group. The weakest staining intensity was found in IUGR. Original magnification x200.

The vitamin D receptor (VDR) was assessed as part of the cellular vitamin D metabolism pathway. There was a significant difference in mean VDR staining levels between the four groups. The normal pregnancy group had higher mean VDR staining levels compared to the other three groups. Late-onset and early-onset preeclampsia groups had lower mean values of VDR staining compared to the normal pregnancy group. Post hoc tests showed a significant difference between normal pregnancy and IUGR groups (p = 0.029). The results of vitamin D receptor (VDR) in placenta are presented in Table 4 and Figs 1 and 2.

**Table 4:** Placental vitamin D receptor (VDR) staining level.

Characteristic VDR	Group					
	Normal	Late-Onset PE	Early-Onset PE	IUGR	Total	p-value
	(n = 10)	(n = 11)	(n = 9)	(n = 10)	(n = 40)	
Stained trophoblast (proportion)	0.71 (0.15)^a^	0.63 (0.17)	0.65 (0.15)	**0.52 (0.11)**^a^	0.63 (0.16)	**0.045**^#^
Positivity (relation strong vs weak/no staining)	0.62(0.69)	0.55(0.96)	0.65 (0.75)	0.28 (0.58)	0.52 (0.75)	0.708^#^

Data are shown as mean (standard deviation) VDR = Vitamin D receptor;

^#^One-way ANOVA test;

^$^Kruskal-Wallis Test;

^a^: post hoc test between normal pregnancy and IUGR (p = 0.029)

**Fig 3:**
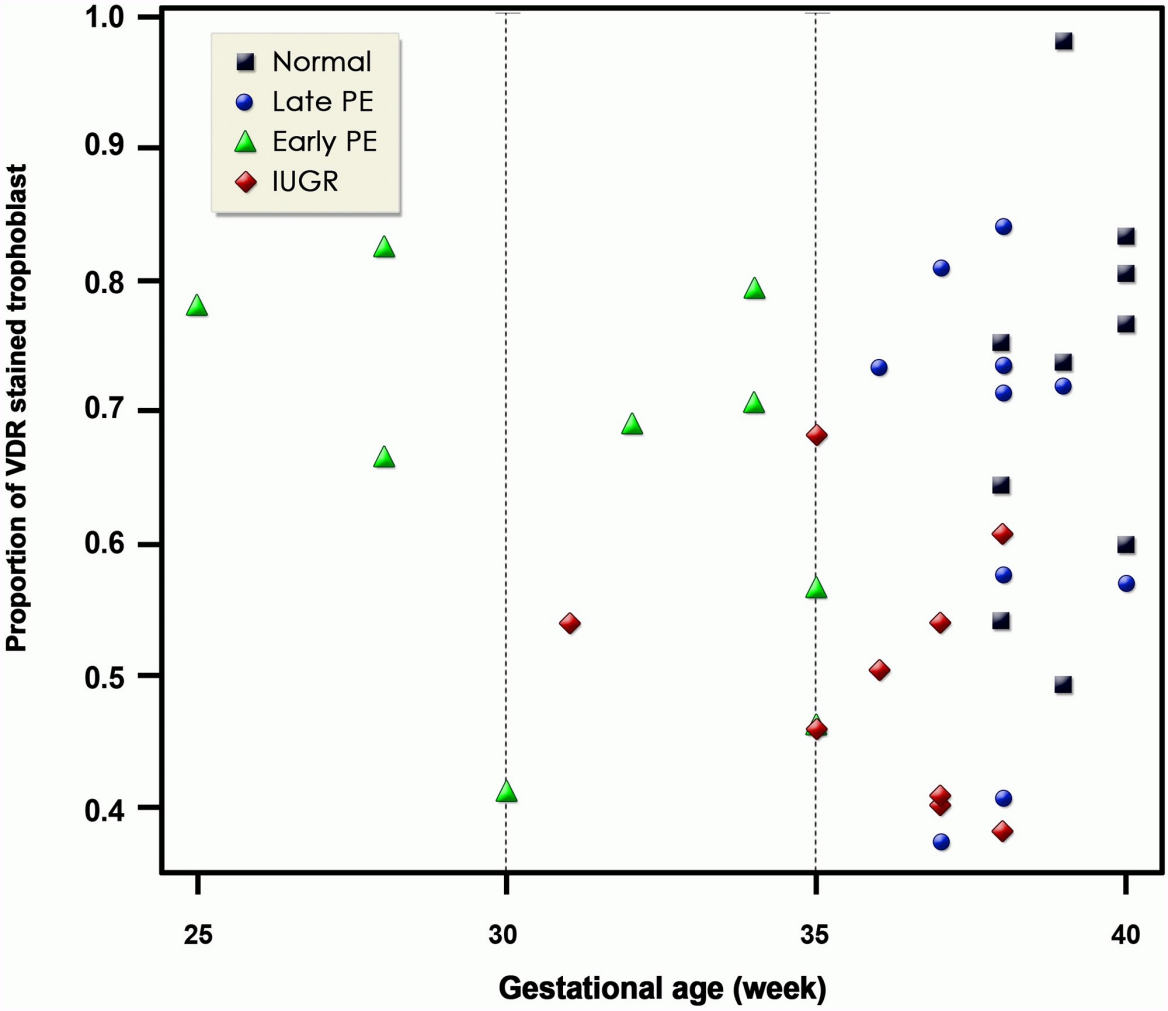
Distribution of placental VDR staining level related to gestational age. There was no significant difference of staining level related to gestational age.

VDR staining levels did not show any correlation to gestational age as depicted in Fig 3.

Correlation tests of 25(OH)D levels in maternal blood and vitamin D receptor staining levels in the placenta were performed in order to explore the activity of VDR. There was a significant difference between 25(OH)D levels in maternal blood and placental tissue VDR with parameters of stained trophoblasts among the four pregnancy groups with a weak correlation (r = 0.38; p = 0.015) as shown in Table 5.

**Table 5:** Correlation between serum maternal 25(OH)D and vitamin D receptor (VDR) staining levels.

Variabels	Group				
	Normal	Late-Onset PE	Early-Onset PE	IUGR	Total
	(n = 10)	(n = 11)	(n = 9)	(n = 10)	(n = 40)
Stained trophoblast (proportion) for VDR	-0.07 (p = 0.855)	0.49 (p = 0.125)	-0.03 (p = 0.932)	0.12 (p = 0.751)	**0.38** **(p = 0.015)**
Relation strong vs weak/no staining for VDR	0.06 (p = 0.881)	0.56 (p = 0.077)	-0.07 (p = 0.865)	-0.20 (p = 0.577)	0.21 (p = 0.190)

Data are shown as Spearman Correlation Coefficient.; VDR = Vitamin D receptor

Correlation analysis was performed between the maternal vitamin D level and VDR staining levels in the placenta. The analysis between these two was performed due to their involvement in the vitamin D metabolism. Since the data was not normally distributed, Spearman Correlation Analysis was used. The coefficient of correlation, showing the power of correlation, was below 0.4 and thus considered to show a weak correlation. At the same time, the p value between groups was significant for stained trophoblast. The interpretation is that there was a weak correlation but significant difference between groups for stained trophoblast.

### Trophoblast survival capacity and placental VDR

**Fig 4:**
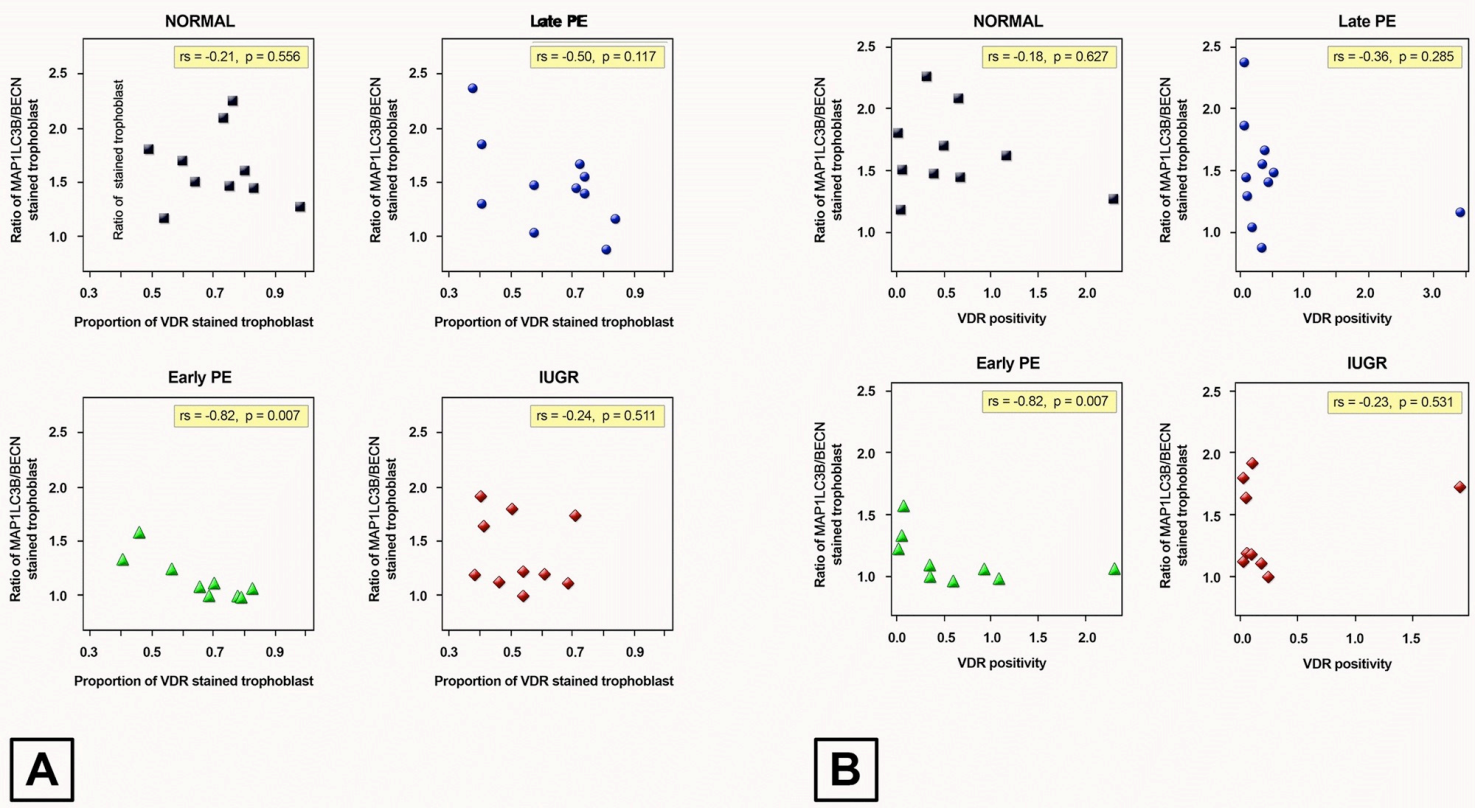
Correlation analysis between the MAP1LC3B/BECN1 ratio and placental VDR staining for (A) stained trophoblast and (B) positivity. The results show that there was a significant and strongest correlation between the survival marker and placental VDR in the early onset preeclampsia group.

Correlation between the MAP1LC3B/BECN1 ratio as cellular survival marker and intracellular VDR in villous trophoblast in the four study groups was performed. All groups showed a negative correlation, with the normal pregnancy group showing the strongest correlation, followed by the IUGR group, the late-onset preeclampsia group and the early-onset preeclampsia group. Furthermore, there was a strong and significant correlation between the MAP1LC3B/BECN1 ratio as survival marker and VDR levels in the early-onset preeclampsia group (Table 6 and Fig 4).

**Table 6:** Correlation between the ratio MAP1LC3B/BECN1 and placental VDR staining level.

Variable	Group				
	Normal	Late-Onset PE	Early-Onset PE	IUGR	Total
	(n = 10)	(n = 11)	(n = 9)	(n = 10)	(n = 40)
**Ratio MAP1LC3B/BECN1**					
Stained trophoblast in VDR	-0.21 (p = 0.556)	-0.50 (p = 0.117)	**-0.82** **(p = 0.007)**	-0.24 (p = 0.511)	-0.27 (p = 0.096)
Positivity in VDR	-0.18 (p = 0.627)	-0.36 (p = 0.285)	**-0.68** **(p = 0.042)**	-0.23 (p = 0.531)	-0.19 (p = 0.230)

Data analysed by Spearman Correlation; VDR = Vitamin D Receptor

Correlation analysis was performed between the MAP1LC3B/BECN1 ratio as survival marker and VDR staining levels in the placenta. Since the data was not normally distributed, Spearman Correlation Analysis was used.

The coefficient of correlation, showing the power of correlation, was below 0.4 for all groups and thus considered to show a weak correlation. At the same time, the early-onset preeclampsia group showed a coefficient above 0.6 and was considered strong and the p-value showed a significant correlation. Thus, it seems as if the survival marker is mostly regulated by VDR as part of the vitamin D metabolism. The results show that early onset PE reveals the strongest correlation and significance value pointing to the fact that early onset PE has the poorest survival capacity and the lowest VDR staining level, linked to the most severe clinical features.

## Discussion

### Conceptual thinking

The production of angiogenic factors and placental proteins by the placenta reflects the physiological processes occurring during pregnancy.[7] Both angiogenic factors and LGALS13 are assumed to play roles in trophoblast differentiation and invasion.[8,28–30] Defective placentation will thus result in changes and fluctuative amounts of these markers released into the circulation. The quality of trophoblast differentiation is shown by LGALS13 and the ratio of sFLT1/PGF as angiogenic factors.

**Fig 5:**
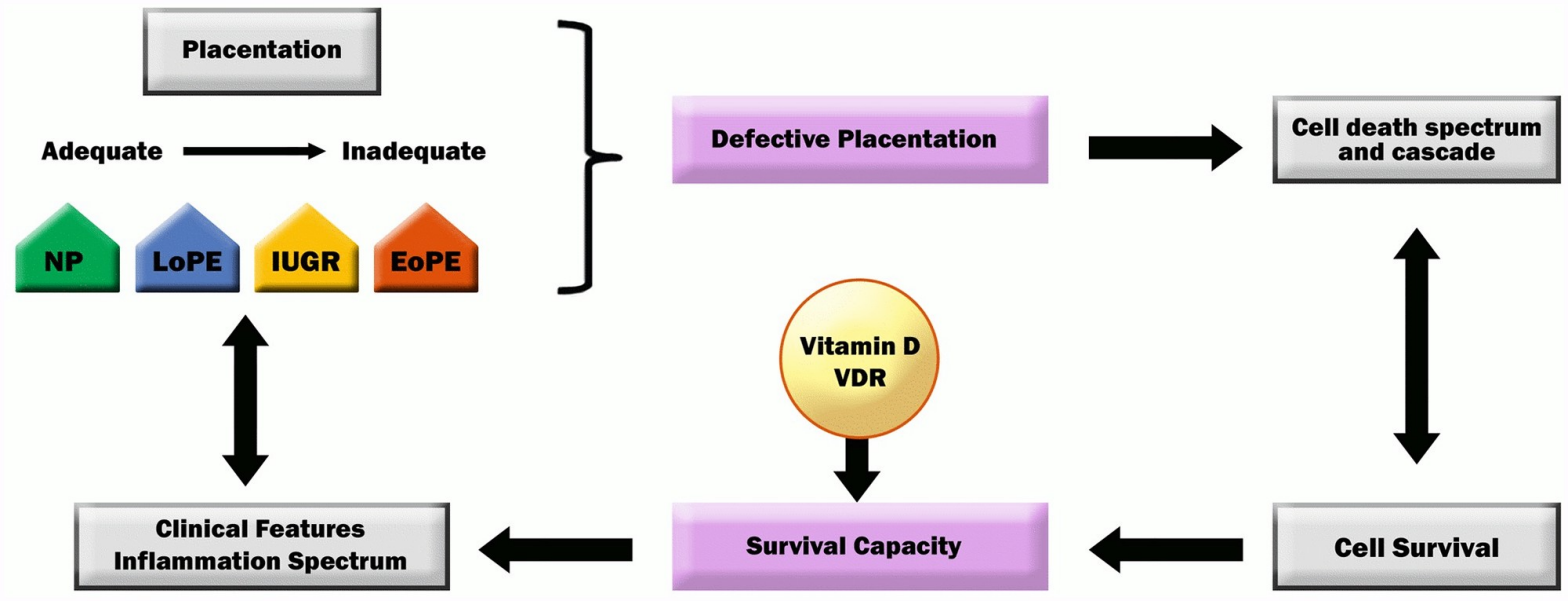
Conceptual thinking of defective placentation in early pregnancy and placental survival capacity in pregnancy pathologies. Defective placentation will induce changes in the cell death spectrum. The latter are counteracted by the cellular survival capacity as adaptation mechanism which is also regulated by micronutrients such as vitamin D and its vitamin D receptor. NP: normal pregnancy, LoPE: late-onset preeclampsia, EoPE: early-onset preeclampsia, IUGR: intra-uterine growth restriction, VDR: vitamin D receptor.

Inadequate trophoblast differentiation will result in a cellular death spectrum ranging from apoptosis to necrosis and will subsequently induce inflammation and immunological responses of the mother. In this context, not only cell death occurs but also the cellular survival capacity, autophagy, takes over a major role.[14,31,32] The dual role of autophagy in either cell death or cellular survival has been shown in the trophoblast already.[33–35] The proposed survival capacity marker, the ratio of MAP1LC3B/BECN1 shows the ability of the trophoblast to survive even in the presence of environmental stress.[13] The conceptual thinking of this study is shown in Fig 5.

### Defective placentation and cellular survival

Defective placentation is a condition occurring as a result of inadequate trophoblast differentiation in early pregnancy. Extravillous and villous trophoblast subtypes do not successfully differentiate and mature and thus the transformation of spiral arteries as well as the placenta proper fail to adequately support the healthy continuity of pregnancy.[7,36] The question arises where in this spectrum of failure diseases and syndromes develop such as preeclampsia and IUGR. Here we hypothesize that the cellular survival capacity based on autophagy plays an important role in cell death and cellular survival in the above syndromes.

The results of failure of trophoblast differentiation can be visualized by the altered ratio of sFLT1/PGF and altered LGALS13 release. The angiogenic factors sFLT1 and PGF play roles in angiogenesis, while LGALS13 is released from the syncytiotrophoblast and is involved in regulating the maternal blood pressure.[8,29,37] The results of this study showed normal sFLT1/PGF ratios in normal pregnancy and IUGR cases while there were higher values in late and early-onset preeclampsia. This is in line with the data from the PROGNOSIS study where the authors decribed a cut-off value of 38 for the sFLT1/PGF ratio.[29] Interestingly, the data on LGALS13 did not show an increase in cases with preeclampsia, different to what has been described before.[8,30]

The trophoblast survival capacity was shown using the MAP1LC3B/BECN1 ratio. There was a significant difference in the mean values of the MAP1LC3B/BECN1 ratio among the four study groups. The early-onset preeclampsia group had a significantly lower mean value than the control group. This could be interpreted that early-onset preeclampsia had the worst ability to maintain cell survival compared to all other groups.[13] However, it cannot be ruled out that gestational age may have an impact here as directly age-matched controls are missing.

### Vitamin D in pregnancy

Vitamin D is a *secosteroid* hormone which is known to have important roles in many physiological processes, such as regulation of cell proliferation and differentiation processes, immunomodulation, vascular biology, and placental metabolism and function.[38] A meta-analysis recently stated that vitamin D deficiency in pregnancy increases the risk of pathological pregnancies, including increased risks for preeclampsia (2.09), preterm labor (1.58), and low birth weight (1.52).[39] The role of vitamin D in immunological processes at the fetal-maternal interface is the reason for studies on vitamin D in preeclampsia.[40]

The high number of preeclampsia cases in Indonesia is suspicious due to the known deficiency of vitamin D in the maternal circulation. Regardless of the geographical position over the equator belt with sunlight all year, we assume there any other factors contributing to this deficiency. The main factor that is assumed is the reduced consumption of vitamin D rich nutrients in the daily nutrition of pregnant women. Looking at the 25(OH)D levels in maternal blood, only the control group presented normal levels above 32 ng/ml. All pathological groups showed significantly reduced vitamin D levels as well as vitamin D deficiency. This data can be interpreted as a direct correlation between low vitamin D levels and inflammation and cellular survival of the trophoblast in pregnancy pathologies. The results showed that normal pregnancy had normal reference levels and thus seems to have normal physiological cellular processes mediated by vitamin D. These results were in accordance with previous studies showing the increasing risk of preeclampsia in vitamin D deficiency.[41,42]

### Vitamin D receptor (VDR)

As the placenta is the source of one of the extra-renal hydroxylase enzymes, it has a role in vitamin D metabolism. In addition to this, the placenta also expresses the vitamin D receptor that is involved in vitamin D signaling in trophoblast.[43]

Looking at the staining percentage in villous trophoblast, there was a significant difference in placental vitamin D receptor (VDR) staining between the four study groups. The IUGR group showed significantly lower VDR expression compared to the other groups. This is consistent with a previous study showing low VDR levels in IUGR.[19] In preeclampsia cases, VDR staining was higher compared to IUGR but lower compared to normal cases. In the correlation analysis between vitamin D levels of maternal blood and placental VDR staining in villous trophoblast, there was a significant difference between the study groups. The results of this VDR analysis showed the dynamic activity of VDR as response to vitamin D levels in circulation. This suggests a mechanism of up and down-regulation of the receptor to adapt to environment stress.[16] It shows the up-regulation mechanisms in the preeclampsia groups, while in IUGR the down regulation shows the phenotypic changes of the placenta known as compromised placenta in IUGR.[44]

### Placental VDR and trophoblast survival capacity

The correlation test between the trophoblast survival marker, the MAP1LC3B/BECN1 ratio, and placental VDR revealed negative correlations in all study groups. This may explain the inadequacy of cellular nutrition for vitamin D, as systematically reflected by the vitamin D receptor (VDR) and thus the low cellular survival in the pathological cases with vitamin D deficiency. Moreover, the early-onset preeclampsia group showed the significantly strongest negative correlation compared to the other study groups. This result may point to the fact that the vitamin D receptor may play a role in the cellular survival capacity, especially in early-onset preeclampsia.

The cellular survival capacity can be integrated into the concept of cellular homeodynamics where the survival capacity can be seen as cellular buffer counteracting environmental or developmental stress.[10] This buffer is supported by hormetins such as nutrition including vitamin D.[17] Vitamin D acts on the genomic and non-genomic level to influence cellular processes and thus may an important regulator in the dynamic processes of pregnancy. Vitamin D and its cellular binding partner VDR may thus have a direct impact on the survival capacity of trophoblast as seen in Fig 5.

## Limitations

There were several limitations encountered in this study. First, this pilot study was carried out in a cross-sectional design with small numbers of samples. Additionally, only one measurement was perfomed at the time of delivery. Due to observations of this study that there is a crosstalk between autophagy and vitamin D as survival nutrient, which in turn should stimulate a larger longitudinal and experimental study. Second, the assessments were only performed in a static manner using immunohistochemical staining of autophagy proteins MAP1LC3B and BECN1 and vitamin D receptor. Third, placental tissue was collected at the end of pregnancy which did not allow for real time dynamic interpretation of pregnancy and cellular homeodynamics. Hence, the interpretation should be cautious but comprehensive to capture the multiple facets of this dynamic process.

## Conclusion

The placenta as a pregnancy-specific organ has mechanisms of adaptation and compensation to counteract environmental stress. This becomes obvious looking at the ratio of MAP1LC3B/BECN1 as survival marker, which can be used to describe the survival capacity of villous trophoblast. Defective placentation in preeclampsia is negatively supported by vitamin D deficiency, which should as act as major biological buffer of cellular homeodynamics. Vitamin D and its cellular binding component vitamin D receptor (VDR) might play a role in the trophoblast survival capacity particularly in preeclampsia.

## Supporting information

S1 File (XLSX)

S2 File (XLSX)
